# Microstructure, Hardness, and Wear Behavior of Layers Obtained by Electric Arc Hardfacing Processes

**DOI:** 10.3390/ma18020299

**Published:** 2025-01-10

**Authors:** Sebastian Balos, Danka Labus Zlatanović, Petar Janjatović, Milan Pećanac, Olivera Erić Cekić, Milena Rosić, Srećko Stopić

**Affiliations:** 1 Department of Production Engineering, Faculty of Technical Sciences, University of Novi Sad, Trg Dositeja Obradovica 6, 21000 Novi Sad, Serbia; sebab@uns.ac.rs (S.B.); janjatovic@uns.ac.rs (P.J.); pecanac.milan@uns.ac.rs (M.P.); 2 Department of Production Technology, Technische Universität Ilmenau, 98693 Ilmenau, Germany; danka.labus-zlatanovic@tu-ilmenau.de; 3 Innovation Center, Faculty of Mechanical Engineering, University of Belgrade, Kraljice Marije 16, 11120 Belgrade, Serbia; oeric@mas.bg.ac.rs; 4 Faculty of Mechanical and Civil Engineering in Kraljevo, University of Kragujevac, Dositejeva 19, 36000 Kraljevo, Serbia; 5 “Vinča” Institute of Nuclear Sciences, National Institute of the Republic of Serbia, University of Belgrade, Mike Petrovića Alasa 12-14, 11351 Belgrade, Serbia; 6 IME Process Metallurgy and Metal Recycling, RWTH Aachen University, Intzestraße 3, 52056 Aachen, Germany

**Keywords:** hardfacing, microstructure, microhardness, abrasive wear resistance, shielded metal arc welding

## Abstract

Hardfacing is a welding-related technique aimed at depositing a harder and tougher layer onto a softer, less wear-resistant substrate or base metal. This process enhances the abrasion resistance of the component, increasing its durability under working conditions. A key feature of hardfacing is dilution, which refers to the mixing of the hardfacing layer and the base metal. In this study, shielded metal arc welding (SMAW) was employed to hardface structural steel using chromium carbide vanadium consumables, with results compared to AISI D2 cold-work tool steel. Four different SMAW parameters were tested, and the abrasive test was conducted against SiC discs. Wear rate, represented by the wear loss rate, was correlated to microstructure, scanning electron microscopy, energy-dispersive X-ray spectroscopy, hardness, microhardness, and surface roughness. The results showed that key SMAW parameters, such as welding speed and current, significantly influence wear resistance. Specifically, slower welding speeds and higher currents, which result in greater heat input, led to the increased wear resistance of the deposited layer through the mechanism of the inoculation of larger and harder carbides.

## 1. Introduction

Hardfacing is a deposition technique that can be applied to various base materials, most commonly relatively affordable structural materials, such as steel. The aim of hardfacing is twofold: to restore dimensions, i.e., re-establish the shape and size of the worn or corroded part, or to impart entirely new properties to a newly produced component [1,2].

This technological process can significantly enhance resistance to wear caused by abrasion, impact, adhesion, corrosion, heat, or a combined effect of any of the mentioned properties [3,4]. Thus, hardfacing can improve the service life of fabricated parts and prevent fractures and breakdowns in a cost-effective manner for both maintenance and manufacturing. Maintenance costs are reduced, since hardfaced parts have a longer life and are less prone to failure. Therefore, instead of replacing these failed parts, they can be repaired or regularly maintained through on-site hardfacing, thereby reducing both planned and unplanned downtime. Furthermore, production costs can be lower, since only the surface layer is made of expensive material, while base materials are typically made of low-cost materials that are easy to fabricate and join by welding if needed [5,6,7]. Hardfacing is generally a cost-effective technology and can be performed using existing welding equipment.

The most common welding techniques used for hardfacing include arc welding, gas-shielded arc welding, and powder-based welding. Among arc welding processes, the most common are shielded metal arc welding (SMAW) [8,9] and submerged arc welding [10,11]. Gas-shielded metal arc processes include gas tungsten arc welding (GTAW) [5,6], gas metal arc welding (GMAW), as well as flux cored arc welding (FCAW), and metal cored arc welding (MCAW) [12,13,14]. Finally, for powder-based processes, plasma transfer arc welding (PTAW) [15,16,17] and laser cladding/welding (LC/LW) are the most common, with the latter becoming increasingly attractive due to a drop in cost [15,18].

A wide range of hardfacing techniques corresponds to the diverse welding processes that encompass the majority of industrial joining technologies. Furthermore, this diversity is reflected in the variety of materials that can be deposited, which in turn, influences applications in various industries, such as agriculture, mining, nuclear, construction, general manufacturing, and railways. Even the defense industry has benefited from hardfacing, as several studies have explored its potential to enhance ballistic protection against kinetic energy projectiles [19,20].

Hardfacing consumables can generally be categorized into four main groups, closely corresponding to their base alloy: cobalt-based, nickel-based, tungsten-based, and iron-based [9,21]. Cobalt-based alloys also contain Cr, W, Ni, Mo, and C, providing good wear resistance combined with corrosion and heat resistance, offering excellent versatility, but at a relatively high cost. Furthermore, Co is considered a critical raw material in the European Union and is often targeted to be fully or partially replaced by Ni. Compared to Ni-based alloys, Co-based hardfacing alloys offer better weldability [22,23,24]. Ni-based alloy deposits (Ni-) combine abrasion resistance (due to additions of C, B, and Nb) with corrosion resistance (from Cr and Al additions), particularly at high temperatures (with W and Mo) [25,26]. Tungsten alloys, often referred to as cemented carbides, consist of tungsten carbide (WC) particles with a binder, mainly Co. These deposits have extreme abrasion resistance due to their very high hardness. However, unlike other high-hardness materials, such as sintered ceramics, these alloys have a Co binder that offsets the brittleness of WC and provides resistance to impact loading [25,27]. On the other hand, there is ongoing interest in partially replacing WC and Co as critical raw materials in the EU [24].

Iron-based alloys are the most cost-effective and widely used hardfacing material. These alloys primarily contain C, Cr, Mo, and other elements, similar to those found in ferrous alloys. Fe-Cr-C hardfacing alloys are predominantly used for parts subject to grinding abrasion combined with light impact, such as digging teeth, conveyor screws, mixer wings, scraper blades, rendering plant equipment, and sand pumps (around 3% C and 30% Cr) [UTP Voestapline WEARstick XD60 brochure, accessed on 19 December 2024; UTP Voestapline WEARstick XD61 brochure, accessed on 19 December 2024]. Alloys containing around 4.5% C and 20–25% Cr are suitable for extreme sliding abrasion, including at temperatures up to 500 °C, due to their extremely high abrasion resistance from the very high content of special carbides based on Mo, V, W, and Nb. Their main applications are in earth-moving equipment, the cement and brick industry, and steel mills for radial breakers and revolving bar screens in sintering plants [UTP Voestapline WEARstick XD65 brochure, accessed on 19 December 2024]. Alloys with around 6.5% C and 25% Cr are suitable for hardfacing parts subjected to heavy abrasion with moderate impact. Typical applications include parts for cement presses, brick presses, refractory press screws, conveyor screws, and mixer blades, with service temperatures up to 450 °C [UTP Voestapline WEARstick XD63 brochure, accessed on 19 December 2024]. Their properties are closely related to their microstructure, where Cr-C or Cr-C-B carbides are mixed with the matrix containing one or more common microstructures in ferrous alloys, such as austenite, ferrite, and martensite [1,25,26]. Besides basic alloying elements, a small amount of Nb can be added. Correa et al. [11] reported positive results regarding the formation of primary NbC carbides randomly distributed in the matrix. These findings, despite the marginally lower hardness of the bulk material, resulted in the increased wear resistance of the SAW deposit. This confirmed an earlier study by Berns and Fischer [1,26]. Singh and Pandey [2] investigated the influence of several passes and SAW current, finding that an increased number of passes and a lower deposition current improved wear resistance. This was attributed to a higher hardness achieved in the third pass compared to the first. Additionally, a lower current resulted in higher cooling rates, leading to a transformation from a pearlitic to a martensitic matrix surrounding the carbides.

The primary aim of this research was to vary the microstructure and evaluate and compare the intensive abrasion resistance of four Fe-Cr-C deposits produced using manual SMAW processes with varying parameters. The performances of these deposits were benchmarked against AISI D2 cold-work tool steel in the heat-treated condition. The obtained wear rate results (weight loss rates) were correlated to the hardness and microhardness of the deposits, microstructures, particularly carbides, and the local chemical composition.

## 2. Materials and Methods

Hardfacing was performed on S235JR structural steel, with dimensions of 100 mm in length, 50 mm in width, and 30 mm in thickness. The 100 × 50 mm area was hardfaced. The chemical composition of the base metal is shown in Table 1. The consumable used was a rutile-coated electrode (Shielded Metal Arc Welding—SMAW process) from the WEARstick XD 60/UTP Ledurit 60 series (Voestalpine, Linz, Austria), belonging to the Fe-Cr-C type (E Fe14), with the nominal composition presented in Table 2. Hardfacing parameters for Specimens 1 to 4 are shown in Table 3, along with the deposit thickness range. The arc length was manually maintained at approximately 3 mm, with no preheating. Prior to the hardfacing process, the electrodes were dried at 300 °C for 2 h. The SMAW process was conducted on an Iskra E10 device (Iskra, Ljubljana, Slovenia). The hardfacing direction was longitudinal, with a push/forward orientation of the electrode, and the width was covered using stringer beads.

The reference material, which served the base material for the deposited material, was a rolled homogeneous plate made of a cold-work tool steel of the AISI D2 type in a heat-treated, hardened condition. This was selected as an alternative to FeCrC hardfacing on structural steel for application in rendering mills. In this application, a combination of wear and impact is expected, which is why the cold-work FeCrC alloy was chosen as the hardfacing material. This specimen was designated as Specimen D2. The chemical composition is presented in Table 4. Heat treatment involved austenitization at 980 °C, quenching in oil, and tempering at 450 °C to achieve a uniform hardness of 58 HRC. Hardness was measured using an HP-250 Rockwell hardness testing device (WPM-Werkstoffpruefmachinen, Leipzig, Germany).

The wear test was conducted on a customized metallographic laboratory polishing machine, the DP-U2 (Struers, Bellerup, Denmark). The customization involved replacing the polishing wheel with SiC grinding paper, which had a grit size of P100. According to the FEPA (Federation of European Producers of Abrasives), the corresponding SiC abrasive paper particle size is 162 µm, with a loading of 1500 g. Basic surface roughness parameters of the counterbody, based on five measurements, were as follows: Ra (arithmetic mean roughness value) of 3.261 ± 0.237 µm and Rz (maximum height of the profile) of 35.825 ± 3.415 µm. The specimens were mounted in a brass holder and placed in a Struers PdM-Force (Struers, Bellerup, Denmark) specimen mover–rotator. Prior to each wear test, each of the five tested specimens was ground using a full set of SiC abrasive papers: P100, P150, P240, P360, P500, P600, P800, P1000, P1500, and P2000.

The abrasive paper base in the wear testing spindle was set to a speed of 250 rpm, with a wear time of 60 s. The specimen axis was 70 mm away from the centerline of the spindle, giving a sliding distance of 109.9 m. The wear test was performed in a water environment at 15 °C, with a constant flow rate maintained at 100 mL/min. Specimen mass was measured using a Type 2615 analytic balance (Tehtnica, Zelezniki, Slovenia) with an accuracy of 0.1 mg, before and after the wear test. The results were calculated as the average weight loss rate of the five specimens. After wear testing, surface roughness was measured using the SJ-301 (Mitutoyo, Kawasaki, Japan) device, with the average for all five tested specimens reported. The measurement parameters were set to λc = 0.8 × 5 mm and λs = 2.5 mm, and the Gaussian filter method was applied with a tip radius of 2 µm and a tip angle of 60°. Two roughness parameters were measured, Ra and Rz.

The metallographic examination was conducted using a light microscope (LM) Orthoplan (Leitz, Wetzlar, Germany), equipped with an ocular micrometer to measure the heat-affected zone (HAZ) width. Additionally, metallographic examination and chemical composition analysis of phases were carried out using a JSM-6460LV scanning electron microscope (SEM) equipped with an Inca Microanalysis system (Oxford Instruments, Abingdon, UK) for energy-dispersive X-ray spectroscopy (EDS), to evaluate the chemical composition of different phases. Prior to SEM analysis, the specimens were coated with gold using the Leica SCD-005 apparatus (Ball-Tech, Leica—Leitz, Wetzlar, Germany). Metallographic preparation before microscopic examination included grinding with SiC abrasive papers (P150, P240, P360, P500, P600, P800, P1000, P1500, and P2000), polishing with 6, 1, and ¼ μm diamond suspensions, and etching the hardfaced layer with Aqua Regia (17% HNO_3_, 50% HCl in glycerol). Base metal in the hardfaced specimens and the reference cold-work D2 tool steel were etched with Nital (3% HNO_3_ in ethanol). SEM was also used to examine the surface topography of worn surfaces.

To evaluate the hardness of the specimens, the Vickers method was applied. The HPO-250 (WPM, Leipzig, Germany). Vickers hardness measurement device was used. The microhardness of specific phases was tested using the Wilson Tukon-1101 (Buehler, Uzwil, Switzerland), with a minimal load of 10 g. Both methods used a dwell time of 15 s. For each hardness test, five measurements were taken, and the average value was reported.

## 3. Results and Discussion

**Figure 1:**
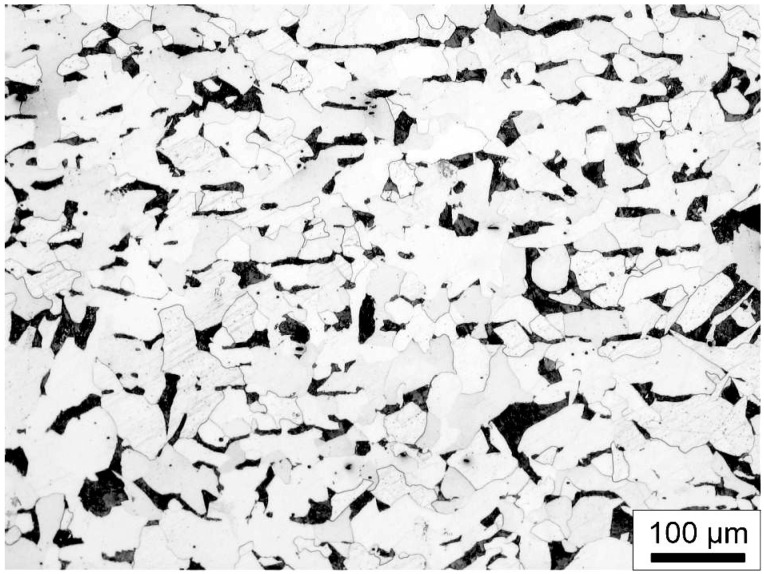
Microstructure of base metal in the form of S235JR structural steel using light microscopy (LM).

The microstructure of the base metal, obtained through light microscopy, is shown in Figure 1. It reveals a structure composed of ferrite and pearlite, which is characteristic of the base metal (structural steel S235JR) to which the hardfacing layer was deposited.

This section is divided into subheadings and provides a concise and precise description of the experimental results, their interpretation, and the experimental conclusions that can be drawn.

**Figure 2:**
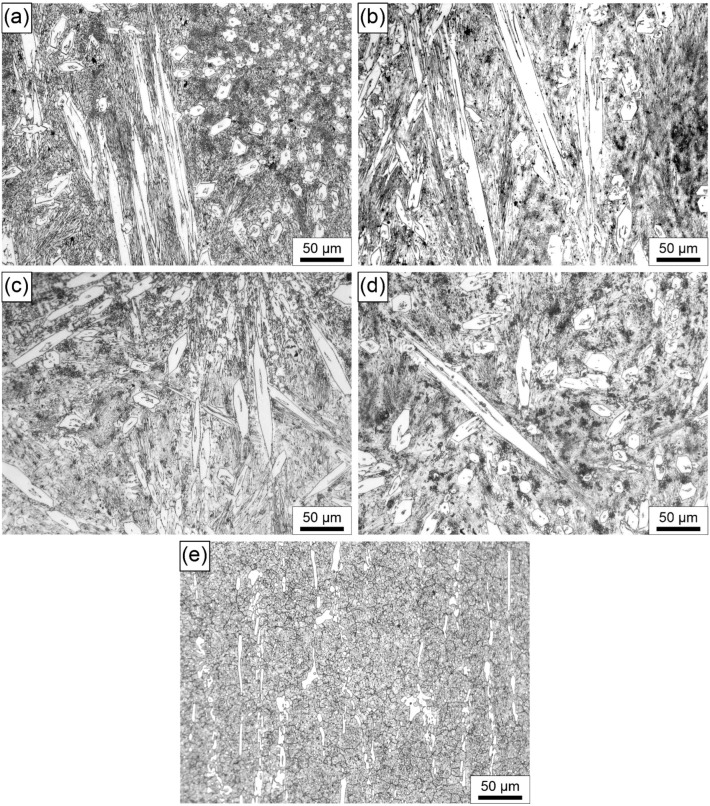
LM images of microstructure of the final layer: (**a**) Specimen 1; (**b**) Specimen 2; (**c**) Specimen 3; (**d**) Specimen 4; (**e**) Specimen D2.

The microstructures of the subsurface finishing layer, which is critical for wear resistance, are presented in Figure 2. The light microscope analysis reveals a similarly shaped white phase, with an approximately hexagonal cross-section and elongated morphology. The growth of these phases, believed to be M_7_C_3_ carbides, occurs in various directions, forming star-shaped formations. Hardfacing alloys of the Fe-Cr-C type rely on large carbides to provide wear resistance. There are no significant differences in the morphology of the white phases observed in Specimens 1 to 4. However, in Specimen 4, these white phases appear larger compared to Specimens 1 to 3. This could be the result of a higher hardfacing current and a lower welding speed, which influence a higher heat input. In turn, higher heat input increases the cooling time, allowing for the more pronounced diffusion of elements like Cr and C, which combine with Fe to form larger complex M_7_C_3_ carbides ((Cr,Fe)_7_C_3_). This can be correlated with the deposit thickness and heat-affected zone, as shown in Table 3, where the deposited layer and heat-affected zone are thickest in Specimen 4. When analyzing Figure 2a–d (Specimens 1 to 4), the minimal dimension of the cross-section should be compared between specimens, as the elongated bright phases are merely a result of the cutting direction during metallographic preparation. To support this, the minimal dimensions of the carbide cross-sections were also measured and reported for Specimens 1 and 4. In Specimen 1, the average carbide dimension was 15 µm, while in Specimen 4, it was 10 µm. On the other hand, in AISI D2 cold-work tool steel (Figure 2e), the microstructure consists of M3C carbides ((Fe,Cr)_3_C), appearing as white strips in the tempered martensitic matrix, which is typical for heat-treated D2 steel, with the heat treatment parameters as explained in the experimental section.

**Figure 3:**
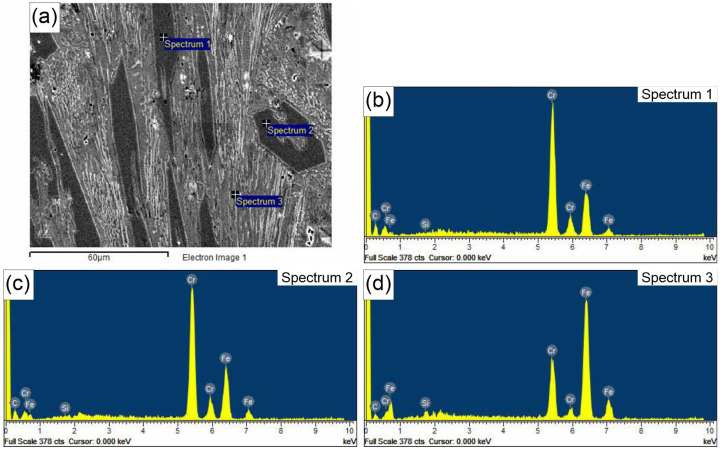
(**a**) SEM micrograph of Specimen 1 EDS spectra of the carbide particles and the eutectic matrix; (**b**) Spectrum 1; (**c**) Spectrum 2; (**d**) Spectrum 3.

**Figure 4:**
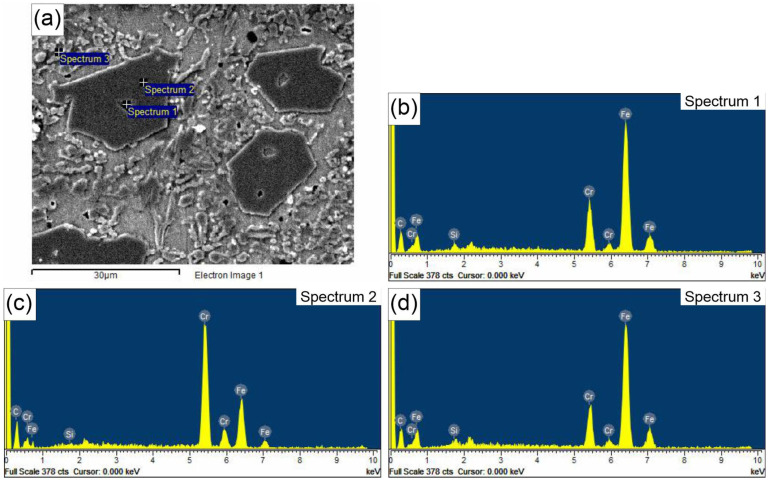
(**a**) SEM micrograph of Specimen 4 EDS spectra of the carbide particles and the eutectic matrix; (**b**) Spectrum 1; (**c**) Spectrum 2; (**d**) Spectrum 3.

In addition to light microscopy, scanning electron microscopy (SEM), specifically energy-dispersive X-ray spectroscopy (EDS), was applied to further describe the microstructures of the hardfaced layers. The results of the EDS analysis are shown in Figure 3 and Figure 4. These reveal a similar composition of primary hexagonal carbides in Specimens 1 and 4 (Spectra 1 and 2 in Figure 3 and Spectrum 2 in Specimen 4). In both specimens, there is a hollow structure within the carbide, as described by Ito et al. [28]. It is proposed that hollow primary carbides form when rapid cooling occurs due to the solidification dynamics during the hardfacing deposition process, which is the main difference between the two specimens analyzed in this work. The key difference is the lower current and welding speed in Specimen 1 compared to Specimen 4. This is evidenced by the core morphology, which is similar to the eutectic matrix consisting of austenite and fine M_7_C_3_ carbides in Specimen 1, and a composition with less Cr in the core compared to the rest of the carbide in Specimen 4 (Spectrum 1). On the other hand, Spectra 3 in Figure 3 and Figure 4 reveal the composition of the eutectic matrix, which contains significantly less Cr and more Fe, resembling the central section of the carbide. This is in agreement with the results obtained by Buytoz [29].

To further explore the properties of the hardfaced layers, the hardness and microhardness of certain phases were measured. Vickers hardness of the bulk material is shown in Table 5. It can be seen that the hardness of the hardfaced layers increases from Specimen 1 to Specimen 4, with Specimen 4 being the hardest. The bulk material hardness of the reference Specimen D2 is the lowest of all the specimens tested in this study.

**Figure 5:**
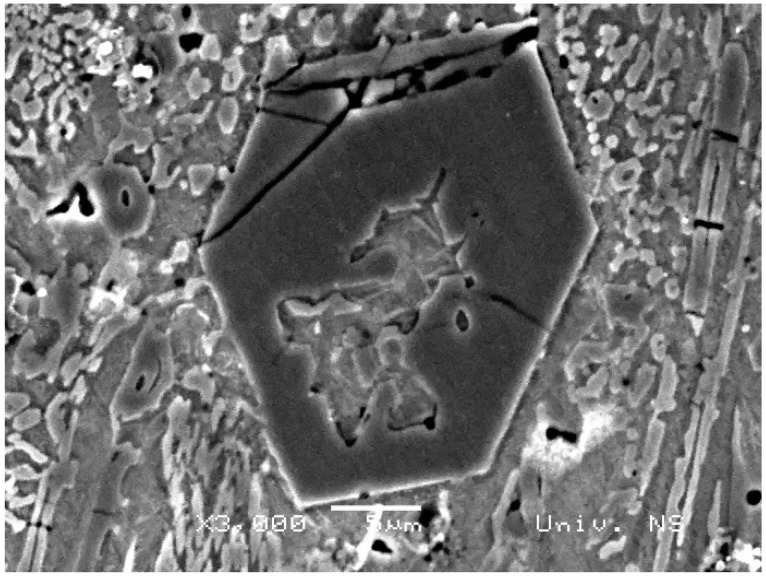
SEM micrograph of cracked M_7_C_3_ carbide in Specimen 1.

Microhardness values for the carbides and eutectic matrix are given in Table 6. Although the same consumable was used, there are notable differences in microhardness, with the highest values obtained in the carbide and eutectic matrix regions of Specimen 4. In the case of M_7_C_3_ carbides, the hardness can be positively correlated with the carbide size. Larger carbides tend to exhibit slightly lower hardness compared to smaller ones, due to the possible presence of flaws, a less homogeneous structure, and a lower density of defects. However, in this study, the opposite result was observed, which can be explained by the large hollow central structure of the carbide filled with material, which is compositionally and morphologically similar to the eutectic matrix. This structure reduces the carbide’s integrity, making it less resistant to deformation. This is supported by Figure 5, which depicts a hollow hexagonal M_7_C_3_ carbide that has cracked in several places. Additionally, a matrix-like structure can be observed in the center of the carbide, which is confirmed by the EDS results presented in Figure 3.

In Specimen D2, significantly lower microhardness values for both the carbides and matrix were obtained. This is consistent with the microstructure of the matrix, which consists of tempered martensite. Furthermore, the carbides are M_3_C, which are not as hard as the M_7_C_3_ carbides found in the hardfaced layers. Moreover, the microhardness values of M_7_C_3_ primary carbides are approximately twice as high as those of the eutectic matrix. The carbide microhardness values for all specimens, presented in Table 6, are consistent with the results reported by Wang et al. [30].

**Figure 6:**
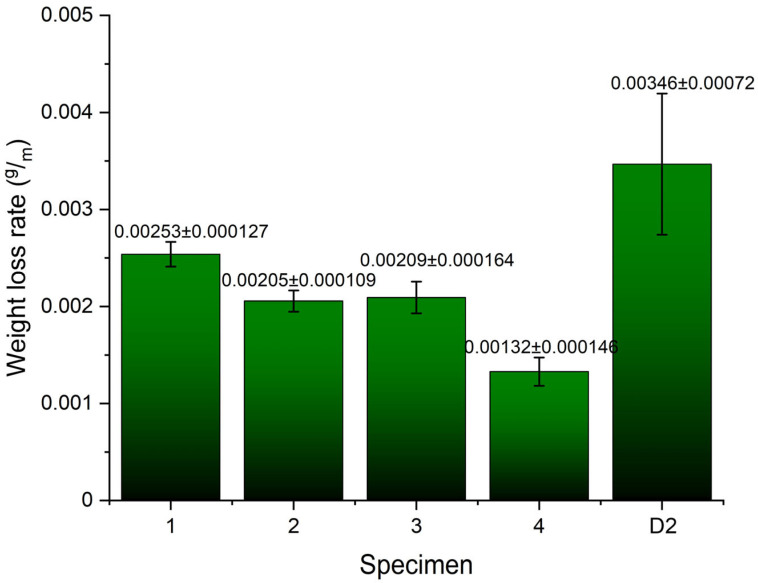
Weight loss rate of tested specimens.

The results of abrasive wear testing, specifically the weight loss rate, are shown in Figure 6. The data suggest that the highest abrasive wear loss rate was observed in Specimen 4. Its weight loss rate is significantly higher than that of Specimen 1, while the weight loss rates of Specimens 2 and 3 are similar. In contrast, the reference Specimen D2 exhibits a significantly lower abrasive wear loss rate compared to any of the hardfaced specimens (Specimens 1 to 4), especially Specimen 4. When compared to Specimen 4, reference Specimen D2 shows about half the weight loss rate. However, it is also evident that the standard deviation in Specimen D2 is the lowest of all the tested specimens, likely due to its relatively homogeneous microstructure, which consists mainly of tempered martensite with a smaller amount of M_3_C carbides. Overall, the higher abrasive wear resistance of the hardfaced specimens can primarily be attributed to the increased amount of M_7_C_3_ carbides, compared to the M_3_C carbides in the reference Specimen D2.

**Figure 7:**
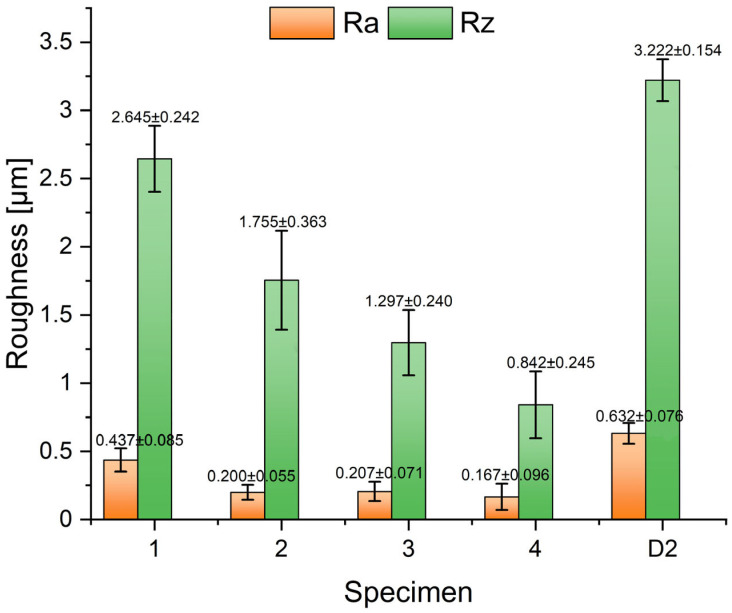
Roughness parameters Ra and Rz for wear tested specimens.

The wear results are further supported by the surface roughness parameter measurements, shown in Figure 7. Based on these results, it can be deduced that the lowest roughness parameters were obtained in Specimen 4, which also exhibited the highest abrasive wear resistance, along with the highest hardness and microhardness. In contrast, Specimen D2 displayed the opposite behavior, exhibiting the highest roughness and the lowest wear resistance. A similar trend of increased roughness compared to Specimen 4 was observed in Specimen 1, which has a similar microstructure but slightly lower hardness and microhardness. The increased surface roughness can be attributed to the cutting action of the hard SiC particles, which abrade the specimen’s surface consisting of carbides and a matrix of lower hardness.

**Figure 8:**
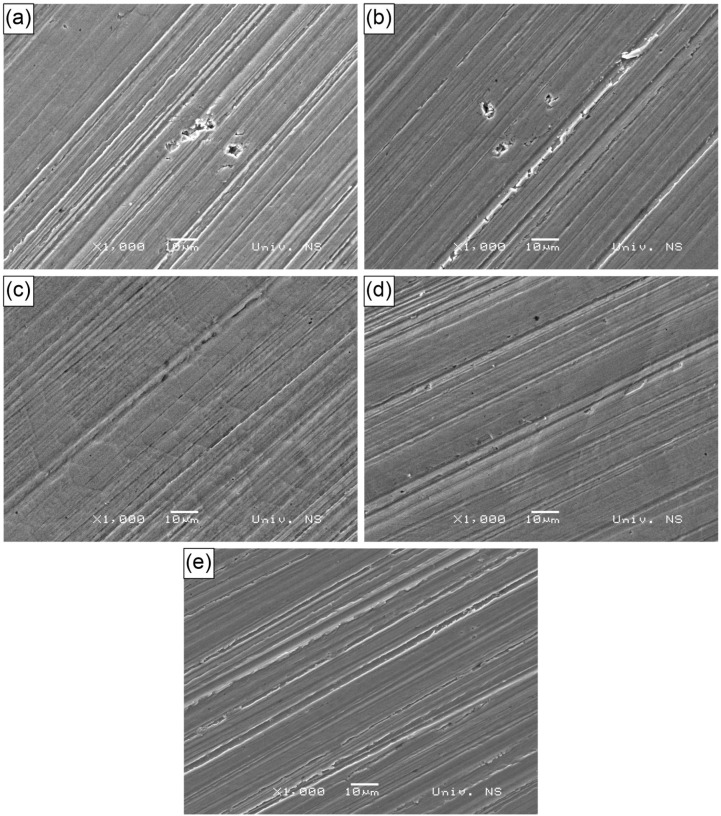
Worn surfaces: (**a**) Specimen 1; (**b**) Specimen 2; (**c**) Specimen 3; (**d**) Specimen 4; (**e**) Specimen D2.

SEM analysis of the worn surfaces is shown in Figure 8a–e. It can be seen that, in Specimens 1 and 2 (Figure 8a,b), ploughing occurs along with mild delamination and the revealing of some pores, ranging from 1 to 3 µm in size, which influence the wear resistance of these hardfaced deposits. Ploughing is a typical mechanism of abrasion. In Specimen 1, a group of craters, likely pores, can also be observed. Porosity may result from a higher cooling rate of these specimens, where the pores form due to the incomplete escape of gases from the melt. In Specimens 3 and 4, ploughing with some delamination occurs, with polygonal carbides visible on the surface. These carbides appear larger in Specimen 4 compared to Specimen 3, which is consistent with the microstructural features of the deposited materials shown in Figure 2c,d. Additionally, in Specimens 3 and 4, small dark dots are present, possibly indicative of minor pores. In all Specimens 1 to 4, areas with flattened asperities are observed. Specimen D2 exhibits more uniform and seemingly more pronounced ploughing and delamination, which is similar to the results presented in [10]. Intensive ploughing is likely the result of intensive abrasion, in accordance with the lower hardness of the material and weight loss rate results compared to Specimens 1 to 4.

## 4. Conclusions

The results obtained within the framework of this study, considering its limitations, may lead to the following conclusions:

- All hardfaced specimens exhibited higher abrasive wear resistance compared to the reference specimen made of D2 heat-treated, cold-work tool steel, due to a higher number of carbides and the overall bulk hardness of the material. An additional effect comes from the higher microhardness of both M_7_C_3_ carbides and the matrix in the hardfaced layers, compared to the microhardness of M_3_C carbides and the martensitic matrix in D2 steel.
- The specimen hardfaced with higher current and lower speed exhibited the highest abrasive wear resistance against relatively hard SiC particles, as well as the lowest roughness parameters.
- High wear resistance is due to the relatively high microhardness of carbides, as well as a larger carbide size compared to other hardfaced specimens obtained with different parameters, i.e., a higher cooling rate.
- Slower cooling influenced the growth of larger M_7_C_3_ carbides, with smaller central hollow sections, which exhibit a higher microhardness compared to the specimen hardfaced with lower current and higher speed (i.e., with a lower cooling time).
- M_7_C_3_ carbides obtained with higher speed and lower current are relatively brittle and prone to cracking just after metallographic preparation, which negatively affects the wear resistance of the parent material. This section is not mandatory but can be added to the manuscript if the discussion is unusually long or complex.
- Weight loss rate results are supported by worn surface analysis. Besides ploughing, the hardfaced specimens exhibit minor delamination and pore formation as a result of incomplete gas escape from the melt. The control specimen was worn uniformly, with intensive ploughing and delamination.

In general, for abrasive wear, hardfacing proved to be more resistant than D2 steel, while in hardfacing, a slower cooling rate—achieved with higher current and slower hardfacing speed—can be beneficial.

## Figures and Tables

**Table 1:** Chemical composition of S235JR (wt.%).

C	Si	Mn	S	Cr	P	Cu	Ni	Fe
0.15	0.18	0.74	0.035	0.02	0.02	0.02	0.05	Balance

**Table 2:** Nominal chemical composition of E Fe14 electrode (wt.%).

C	Si	Cr	Fe
3.0	1.3	29.0	Balance

**Table 3:** Hardfacing parameters for Specimens 1 to 4 (electrode E Fe14).

	Specimen 1	Specimen 2	Specimen 3	Specimen 4
Electrode diameter × length	Ø3.2 × 320 mm			
Arc voltage [V]	25	25	24	24
Welding current [A]	95	95	115	115
Current type	DC+			
Welding speed [cm/min]	14	12	14	12
Preheating	No			
Electrode inclination towards horizontal [°]	80			
Deposit thickness range [mm]	5.5–6	6.5–7	6.5–7	7–7.2
Heat affected zone [mm]	1–1.2	1.4–1.8	1.2–1.6	1.6–1.8

**Table 4:** Chemical composition of the AISI D2 cold-work tool steel (wt.%).

C	Si	Mn	S	Cr	P	Ni	Mo	V	Co	Fe
1.56	0.46	0.34	0.004	12.81	0.024	0.17	0.97	0.90	0.021	Balance

**Table 5:** HV30 hardness of the hardfaced layers and reference AISI D2 steel [kgf/mm^2^].

	Specimen 1	Specimen 2	Specimen 3	Specimen 4	D2
Average values	663 ± 12	707 ± 8	712 ± 13	730 ± 6	640 ± 12

**Table 6:** HV0.01 microhardness of phases within the hardfaced layers [kgf/mm^2^].

	Specimen 1	Specimen 2	Specimen 3	Specimen 4	D2
Carbides, average values	1601.9 ± 54.2	1683.9 ± 79.6	1735.2 ± 128.6	1837.4 ± 49.4	1052.4 ± 64.3
Matrix, average values	851.7 ± 61.8	910.8 ± 80.9	952.1 ± 121.0	997.4 ± 52.6	670.6 ± 50.6

## Data Availability

The original contributions presented in this study are included in the article. Further inquiries can be directed to the corresponding authors.
